# SHARING ROLES IN HOME CARE BETWEEN FAMILY CAREGIVERS AND PAID CAREGIVERS: A NATIONALLY REPRESENTATIVE STUDY

**DOI:** 10.1093/geroni/igae098.1695

**Published:** 2024-12-31

**Authors:** Chanee Fabius, Joseph Gallo, Jennifer Wolff

**Affiliations:** Johns Hopkins University, Baltimore, Maryland, United States; Johns Hopkins Bloomberg School of Public Health, Baltimore, Maryland, United States; Johns Hopkins University, Baltimore, Maryland, United States

## Abstract

Little is known about whether paid caregivers “role-share” with family caregivers in home care, which reflects situations in which members from both groups assist with the same tasks. Home care is often Medicaid-financed, and many care recipients live with dementia. We conducted a cross-sectional analysis of the 2015 National Health and Aging Trends Study. Our sample included 440 community-living older adults receiving paid help with self-care (eating, dressing, bathing, toileting), mobility (getting around inside/outside of the home, getting in/out of bed), or medical care tasks (medication administration, doctors visits). Analyses included Pearson’s chi-square tests and logistic regression models to examine associations between dementia and Medicaid status and experiencing role-sharing or receiving paid care only across individual tasks. Nearly 1.7 million older adults received paid help, of whom half (53%) experienced role-sharing. Older adults whose caregivers shared roles more often lived with dementia but were less often Medicaid-enrolled. In logistic regression models, those living with dementia more often experienced role-sharing in eating (aOR 3.9, p<0.001), bathing (aOR 2.7, p<0.001), dressing (aOR 2.1, p=0.02), toileting (aOR 2.7 p=0.02), and getting around inside the house (aOR 2.8, p<0.01). Those enrolled in Medicaid had greater odds of receiving paid help only in dressing (aOR 2.1, p=0.03), getting around outside (aOR 2.4, p<0.01), and inside (aOR 4.4, p<0.001), as well as doctor visits (aOR 2.8, p<0.01). Findings lay the foundation for interventions that align with national recommendations to increase understanding of and strengthen relationships between family caregivers and paid caregivers.

